# Supplementary material for: Characterizing cell-type spatial relationships across length scales in spatially resolved omics data
Source: Nat Commun. 2025 Jan 3;16:350. doi: 10.1038/s41467-024-55700-1 (PMC11699133; doi:10.1038/s41467-024-55700-1)
Supplement: Supplementary file 3 — Supplementary Software 1 [file 41467_2024_55700_MOESM3_ESM.zip › CRAWDAD/plots/seqfish/2_slideseq_trends_heatmap.pdf]

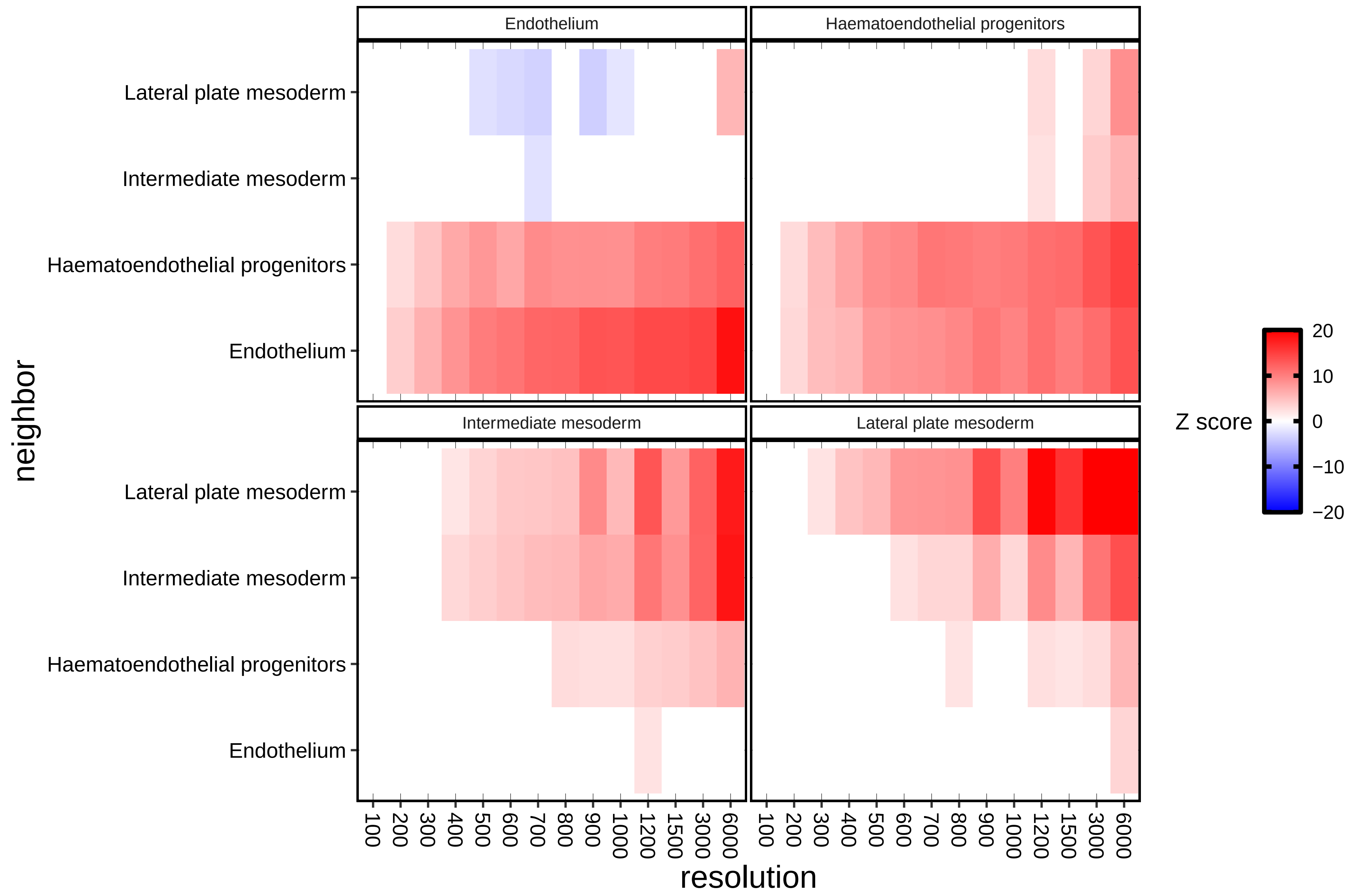

Endothelium
Haematoendothelial progenitors
Intermediate mesoderm
Lateral plate mesoderm
Lateral plate mesoderm
Intermediate mesoderm
Haematoendothelial progenitors
Endothelium
neighbor
100
200
300
400
500
600
700
800
900
1000
1200
1500
3000
6000
resolution
Z score
20
10
0
−10
−20
